# Growth differentiation factor 15 promotes blood vessel growth by stimulating cell cycle progression in repair of critical-sized calvarial defect

**DOI:** 10.1038/s41598-017-09210-4

**Published:** 2017-08-22

**Authors:** Shaoyi Wang, Mengyu Li, Wenjie Zhang, Hongfei Hua, Ningtao Wang, Jun Zhao, Jing Ge, Xinquan Jiang, Zhiyuan Zhang, Dongxia Ye, Chi Yang

**Affiliations:** 1 Department of Oral Surgery, Ninth People’s Hospital, Shanghai Jiao Tong University School of Medicine, Shanghai Key Laboratory of Stomatology, National Clinical Research Center of Stomatology, Shanghai, China; 2 Department of Prosthodontics, Ninth People’s Hospital, Shanghai Jiao Tong University School of Medicine, Shanghai Key Laboratory of Stomatology, National Clinical Research Center of Stomatology, Shanghai, China; 3 Department of Orthodontics, Ninth People’s Hospital, Shanghai Jiao Tong University School of Medicine, Shanghai Key Laboratory of Stomatology, National Clinical Research Center of Stomatology, Shanghai, China; 4 Oral Bioengineering Lab/Regenerative Medicine Lab, Shanghai Research Institute of Stomatology, Ninth People’s Hospital, Shanghai Jiao Tong University School of Medicine, Shanghai Key Laboratory of Stomatology, National Clinical Research Center of Stomatology, Shanghai, China; 5 Shanghai Research Institute of Stomatology, Ninth People’s Hospital, Shanghai Jiao Tong University School of Medicine, Shanghai Key Laboratory of Stomatology, National Clinical Research Center of Stomatology, Shanghai, China

## Abstract

Repair of large bone defects remains a challenge for surgeons, tissue engineering represents a promising approach. However, the use of this technique is limited by delayed vascularization in central regions of the scaffold. Growth differentiation factor 15(GDF15) has recently been reported to be a potential angiogenic cytokine and has an ability to promote the proliferation of human umbilical vein endothelial cells(HUVECs). Whether it can be applied for promoting vascularized bone regeneration is still unknown. In this study, we demonstrated that GDF15 augmented the expression of cyclins D1 and E, induced Rb phosphorylation and E2F-1 nuclear translocation, as well as increased HUVECs proliferation. Furthermore, we also observed that GDF15 promoted the formation of functional vessels at an artificially-induced angiogenic site, and remarkably improved the healing in the repair of critical-sized calvarial defects. Our results confirm the essential role of GDF15 in angiogenesis and suggest its potential beneficial use in regenerative medicine.

## Introduction

Due to trauma, infection, and tumor resection or congenital conditions, large bone defects are quite common and pose a substantial clinical burden. Tissue engineering represents a promising approach for bone regeneration. Current approaches in bone tissue engineering are restricted by delayed vascularization in central regions of the scaffold, which results in cell death in the region and ultimately does not support healing of the defect. Therefore large volume bone defects only regenerate through a highly vascularized tissue, and then progressively transforms into bone. Because of this requirement, the exploration of angiogenic cytokine becomes a focus in tissue engineering^1, 2^.

Angiogenic cytokines can induce angiogenesis and implicate neovascularization in the regenerated tissue, then the vasculature supplies nutrients such as oxygen and to facilitate removal of metabolic waste products. Furthermore blood vessels also transports soluble factors and numerous types of cells to the tissues of the body^3–5^. Many of the factors that lead to the normal development of embryonic vasculature are recapitulated during neoangiogenesis in adults^6^. Previous study has demonstrated that angiogenic cytokines could promote angiogenesis in tissue regeneration and also improve osteogenesis at bone defects^3^. However, these cytokines are apparently not sufficient in the blood vessels regeneration. For example, VEGF promotes HUVECs proliferation and has an angiogenic ability, however, VEGF-induced vessels are often leaky and improperly connected to the existing vasculature^7^. The formation of blood vessels is a complex process that requires the coordination of multiple angiogenic factors and coordinated intercellular communication between cells^8^, thus further investigations are still needed to explore the angiogenic cytokine creating a functional vasculature for tissue regeneration.

Growth differentiation factor-15 (GDF-15) is a member of a divergent group within the TGF-β superfamily^9–11^, which is weakly expressed in most tissues under basal conditions but is substantially up-regulated under pathological conditions such as tissue injury and inflammation^12, 13^. Previous investigations revealed that GDF15 induced the expression of the hypoxia inducible factor-1a and the expression of its target genes such as VEGF by the activation of the mTOR signaling pathway^14^. Recently researchers have found that GDF15 could stimulate proliferation of human umbilical vein endothelial cells and promote vascular development, and that GDF15 could increase the expression level of VEGF in a time-and dose-dependent manner^14, 15^. In this regard, GDF15 may be considered as a potential angiogenic cytokine. Nevertheless, whether GDF15 can promote angiogenesis and be applied in bone defect remains unknown.

To address these problems, we here designed a protocol for examining the underlying mechanisms of GDF15 in the process of angiogenesis by employing human phosphorkinase array, immunoprecipitation, real-time PCR, western blotting analysis, and tube formation assay *in vitro*. With respect to ascertaining their angiogenic capability *in vivo*, Matrigel plug perfusion assay was applied in mice and the repair of the rat critical size calvarial defect was performed according to previously reported animal model^16^. In effect, the experiments discussing in this article evaluated the potential application of GDF15 to promote angiogenesis *in vitro* and *in vivo* (Supplementary Fig. S1).

## Results

### GDF15 promotes HUVECs proliferation and cell cycle progression

**Figure 1:**
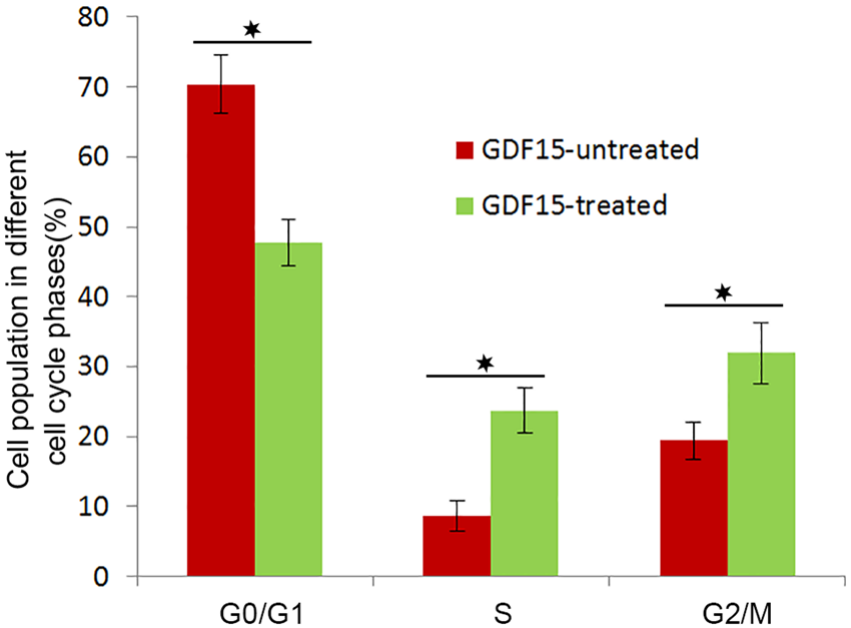
Cell cycle progression of HUVECs treated with GDF15. Serum-starved HUVECs were treated with or without GDF15 for 12 h and incubated in complete medium for 12 h, a larger cell population was observed to progress to the S phase in GDF15-treated cells as compared with untreated cells. The graph shows cell cycle phase distribution from three independent experiments, Y-axis represents cell population in different cell cycle phases.

In order to monitor the effects of GDF15 on HUVECs proliferation, we treated HUVECs in culture with rhGDF15, and found that GDF15 could enhance cell proliferation in a dose dependent manner with low concentration (Supplementary Fig. S2). Then we examined the functional effect of GDF15 on the cell cycle of HUVECs. Serum-starvation for 24 h arrested the majority of cells at the G0/G1 phase, regardless of GDF15 treatment. When serum was supplied to cells, a larger cell population was observe to progress to the S phase in GDF15-treated cells as compared with untreated cells. There was a 2.74-fold increase in the number of GDF15-treated cells in the S phase relative to the control. The data indicate that GDF15 promotes HUVECs cycle progression at the G1 phase and entry into the S stage (Fig. 1).

### GDF15 induces the expression of cyclins D1 and E

**Figure 2:**
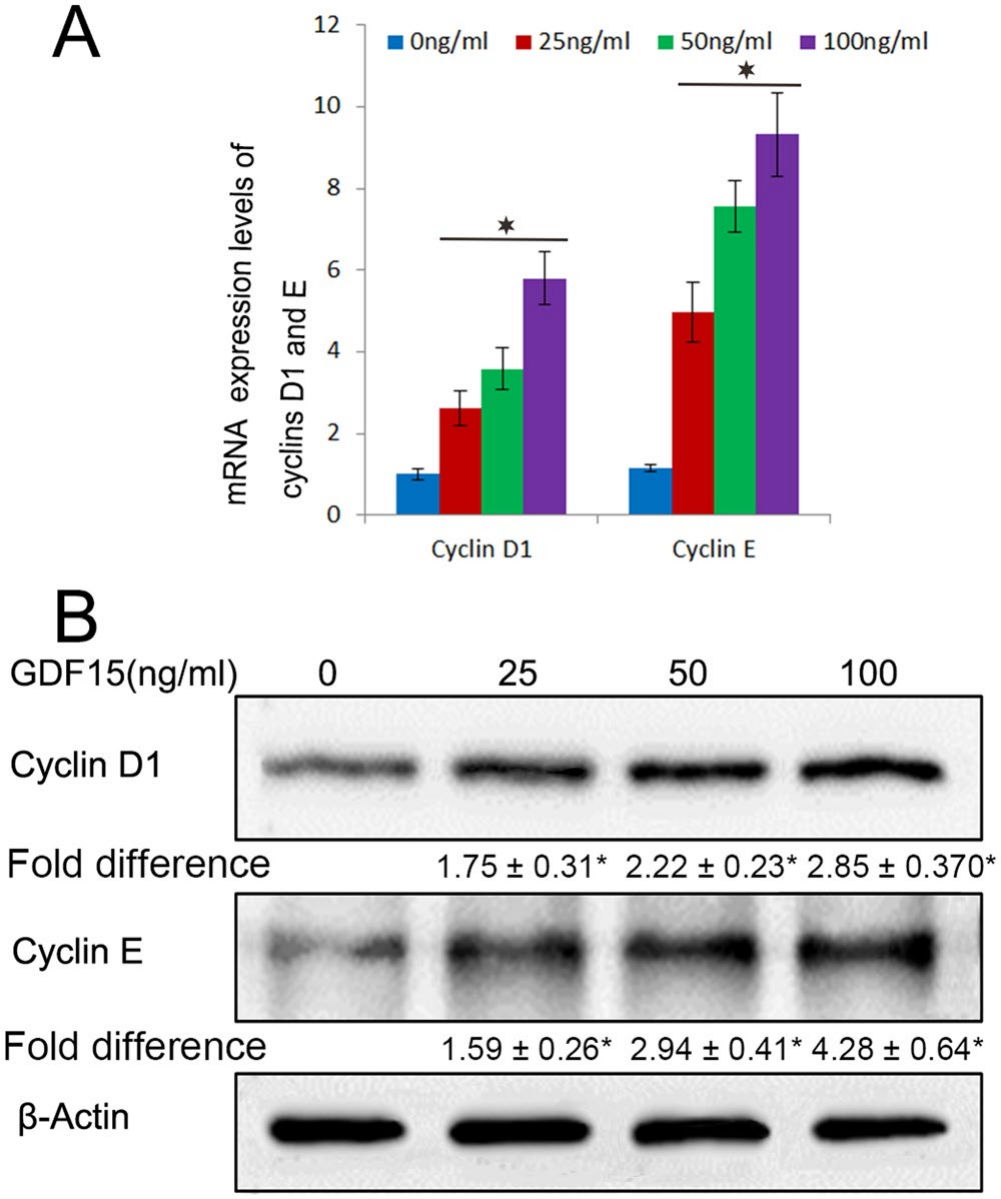
mRNA and protein expression levels of the G1 cyclins, cyclins D1 and E. The mRNA expression levels of the G1 cyclins D1 and E were increasing with 25 ng/ml, 50 ng/ml, 100 ng/mL, and the protein expression was also ascending in a dose dependent manner when cells were treated with GDF15 for 4 h. Numbers under the immunoblot indicate the relative band intensity of protein normalized to the total protein level.

To identify molecules that mediate the cell cycle promoting activity of GDF15, we examined the expression levels of cell cycle machinery components in GDF15-treated and untreated HUVECs for 4 h. We found that the expression of G1 cyclins D1 and E were increased in a dose dependent manner in both mRNA and protein levels (Fig. 2). The results above suggest that GDF15 stimulated the proliferation of HUVECs likely through increased expression of cyclins D1 and E.

### GDF15 increases CDK2,4 activity and induces phosphorylated Rb and E2F-1 nuclear translocation

**Figure 3:**
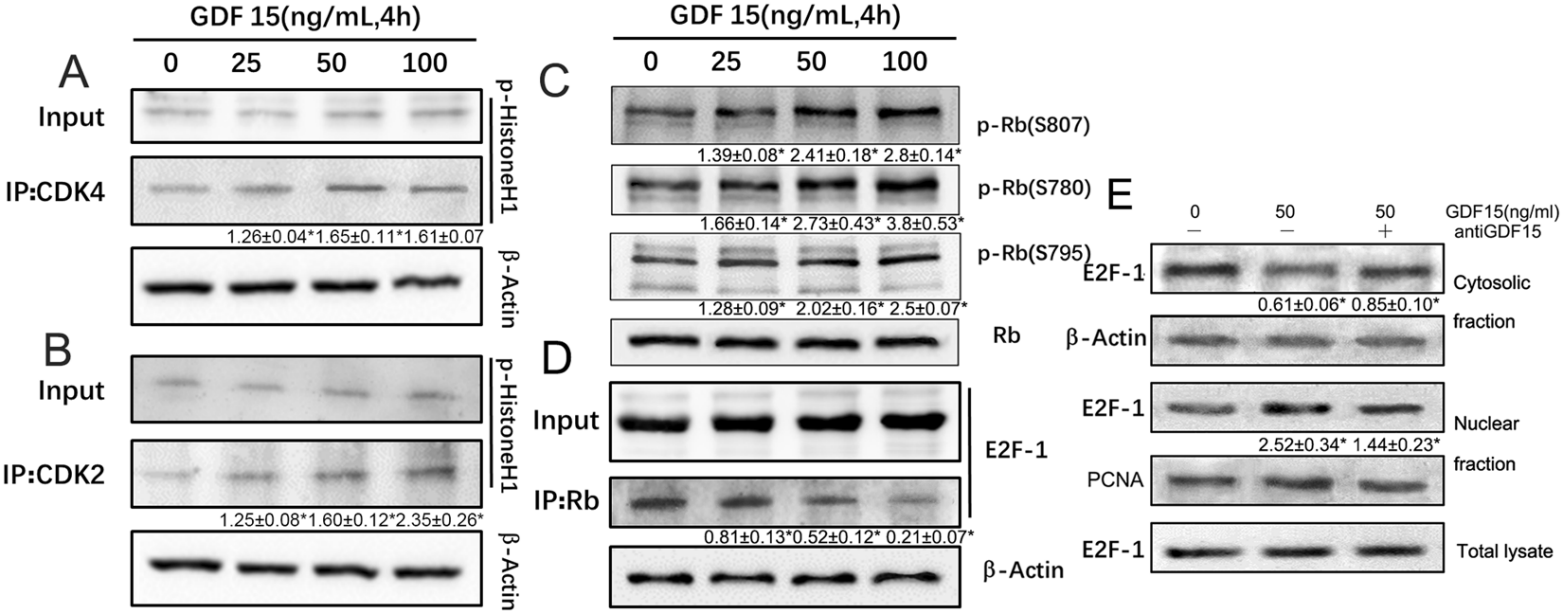
The detection of activities of CDKs, phosphorylation of Rb protein, and nuclear translocation of E2F. After HUVECs were treated with GDF15 for 4 h, CDK2 and CDK4 were immunoprecipitated with anti-CDK2 and anti-CDK4 antibodies, respectively. Co-Immunoprecipitation were subjected to an *in vitro* kinase assay using histone H1 as a substrate (**A**,**B**). Phosphorylation levels of Rb protein in GDF15-treated HUVECs was examined by immunoblotting using antibodies recognizing Rb phosphorylated at Ser-807, Ser-780, or Ser-795 residues (**C**,**D**). The amount of E2F-1 associated with Rb protein was assessed by immunoprecipitation followed by immunoblotting analysis. HUVECs were treated with GDF15 mixed with normal IgG or anti-MIC-1 blocking antibody, then cell lysates were separated into cytosolic and nuclear fractions and the amount of E2F-1 in each fraction was assessed by immunoblotting analysis (**E**). Numbers under the immunoblot indicate the relative band intensity of protein normalized to the total protein level.

Then, we examined the protein kinase activity of CDK4/6 and CDK2, which can be activated by cyclins D1 and E through direct interaction, respectively. The results shown that CDK4 and CDK2 immunoprecipitates both exhibited higher kinase activity toward histone H1 (Fig. 3A and B). We also found that phosphorylated retinoblastoma (Rb) protein, the endogenous substrates for CDK4/6 and CDK2, was increased in a dose-dependent manner, without altering total protein levels (Fig. 3C). As the E2F transcription factor is known to associate with unphosphorylated Rb, but not with phosphorylated Rb. Then we detected the expression of E2F-1 associated with Rb in GDF15-treated cells, which exhibited decreased levels of bound E2F-1 protein to Rb as compared to untreated cells (Fig. 3D). Interestingly, in the nuclear fraction, the E2F-1 level was significantly increased by GDF15, and its cytosolic level was decreased (Fig. 3E). Together, these results indicate that GDF15 induces Rb phosphorylation through enhancing the activity of CDK4/6 and CDK2, then E2F1 release from the Rb–E2F1 complex in the cytosol, resulting in increased nuclear translocation of E2F.

### Signaling pathways regulated by GDF15

To monitor change in HUVECs phosphorylated states in response to GDF15, a proteome profiler assay for human kinases was performed. As shown in Supplementary Fig. 3, after HUVECs were treated with 25,50, and 100ng/mL rhGDF15 for 1 h, AKT, ERK, and JNK were identified whose phosphorylated forms had a tendency to be up-regulated in GDF15-Treated HUVECs. Upregulation of AKT, ERK1/2, JNK was confirmed by Western blot analysis (Supplementary Fig. S3A,B). As AKT, ERK1/2, and JNK signal pathways plays an important role in regulating in cell growth, survival and the inhibition of apoptosis, the above results indicates that these pathways may be involved in promoting the proliferation of HUVECs treated with GDF15.

### Angiogenic ability evaluated by tube formation and Matrigel plug assay

**Figure 4:**
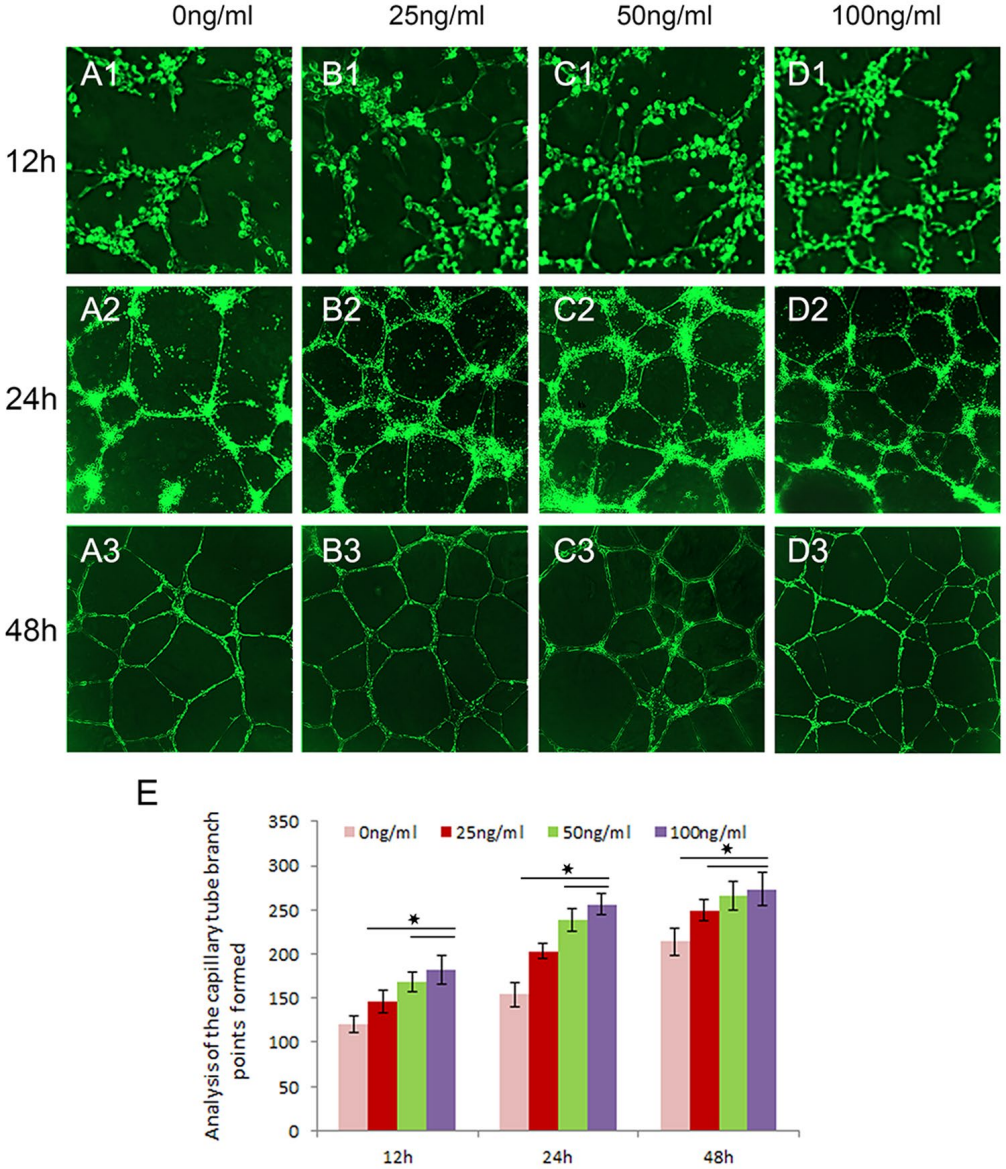
GDF15-induced capillary-like tube formation on Matrigel in vitro. At 12 h, the capillary tube branch points formed in 50 ng/ml and 100 ng/ml groups demonstrated higher than that in the control (**A1**–**D1**). There is no difference between 50 ng/ml and 100 ng/ml groups (**E**). At 24 h, HUVECs were treated with 25 ng/ml, the tube formation of which is lower than that in 50 ng/ml and 100 ng/ml groups (**A2**–**D2**,**E**). At 48 h, tube branch points in the experimental groups were higher than in the control group, and we didn’t observe the difference among the three experimental groups (**B3**–**D3**,**E**).

The angiogenic potential of GDF15 was investigated in a Matrigel assay. Figure 4 shows that GDF15-treated cells generated tube-like structures and networks at 12 h, 24 h, and 48 h. At 12 h, the capillary tube branch points formed in 50 ng/ml and 100ng/ml groups demonstrated higher than that in the control and 25 ng/ml groups. And there is no difference between 50 ng/ml and 100 ng/ml groups (Fig. 4A1–D1). At 24 h, tube formation in the experimental groups is higher than that in the control. HUVECs were treated with 25 ng/ml, the tube formation of which is lower than that in 50 ng/ml and 100ng/ml groups (Fig. 4A2–D2). At 48 h, tube branch points in the experimental groups were higher than in the control group, and we didn’t observe the difference among the three experimental groups (Fig. 4A3–D3).

**Figure 5:**
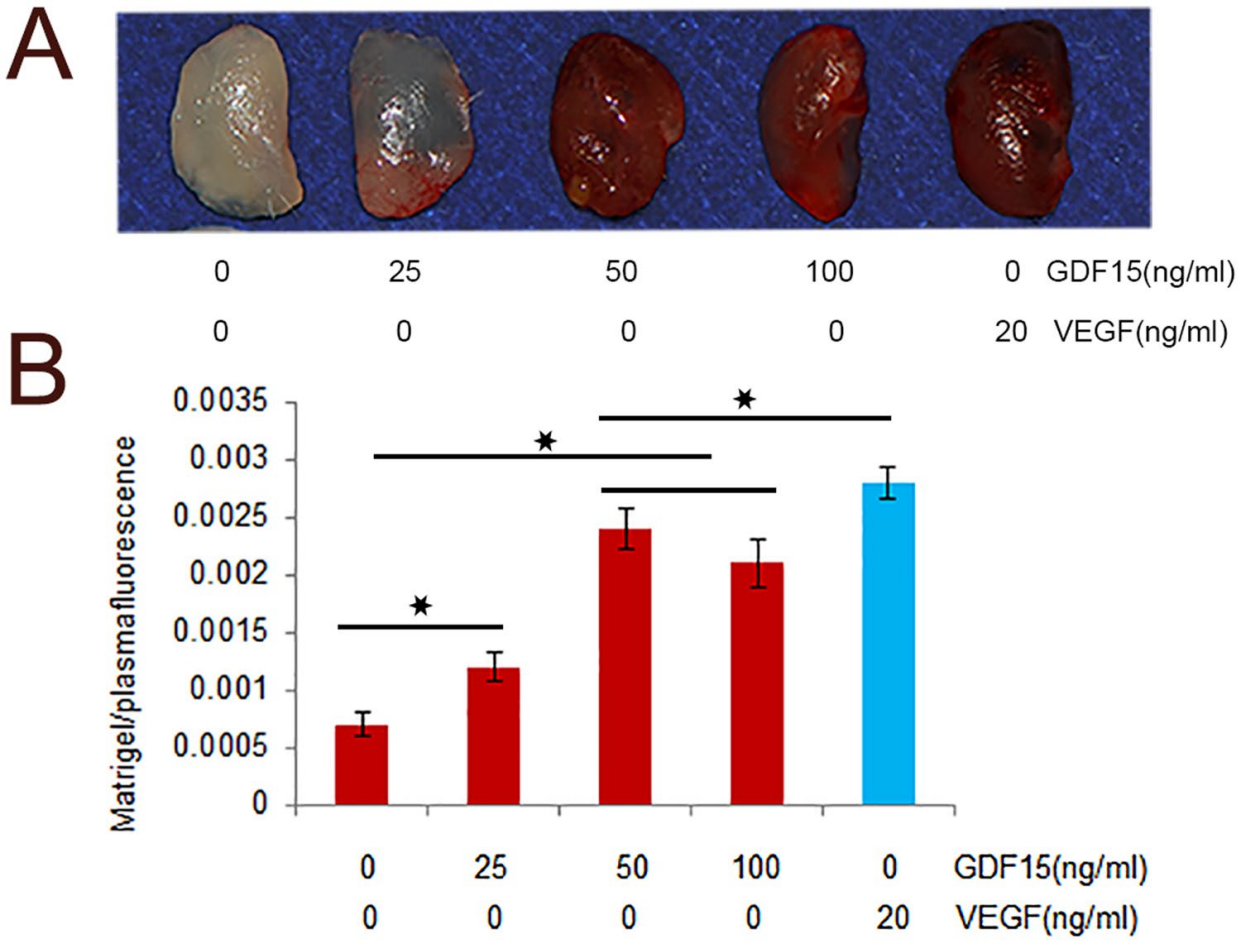
The assessment of angiogenic ability by a Matrigel plug perfusion assay. Representative pictures of Matrigel plug, or plug mixed with 25 ng/ml, 50 ng/ml, 100 ng/ml GDF15 or 20 ng/ml VEGF *in vivo* 2 weeks after grafting (**A**). The ratio between Matrigel plug fluorescence and plasma fluorescence was analyzed, and there was no difference between 50 ng/ml and 100 ng/ml GDF15 groups. This ratio in the three experimental groups was higher than Matrigel alone, but lower than VEGF.

According to Matrigel plug assay, we observed that GDF15 promoted the formation of functional vessels at an artificially-induced angiogenic site. Figure 5A shows gross morphology of Matrigel plugs containing GDF15 and VEGF, demonstrating that functional vasculatures had formed inside the Matrigel via angiogenesis triggered by GDF15. Furthermore, based on the ratio between Matrigel plug fluorescence and plasma fluorescence, we found that the ratios in the 50 ng/ml and 100 ng/ml groups were higher than the other groups, but there was no difference between 50 ng/ml and 100 ng/ml groups. This ratio in the three experimental groups was higher than Matrigel alone, but lower than VEGF group (Fig. 5B). These results confirmed our observations above, and indicated that the angiogenic ablility of GDF15 was induced subcutaneously in the abdominal region.

### Laser Doppler imaging analysis

**Figure 6:**
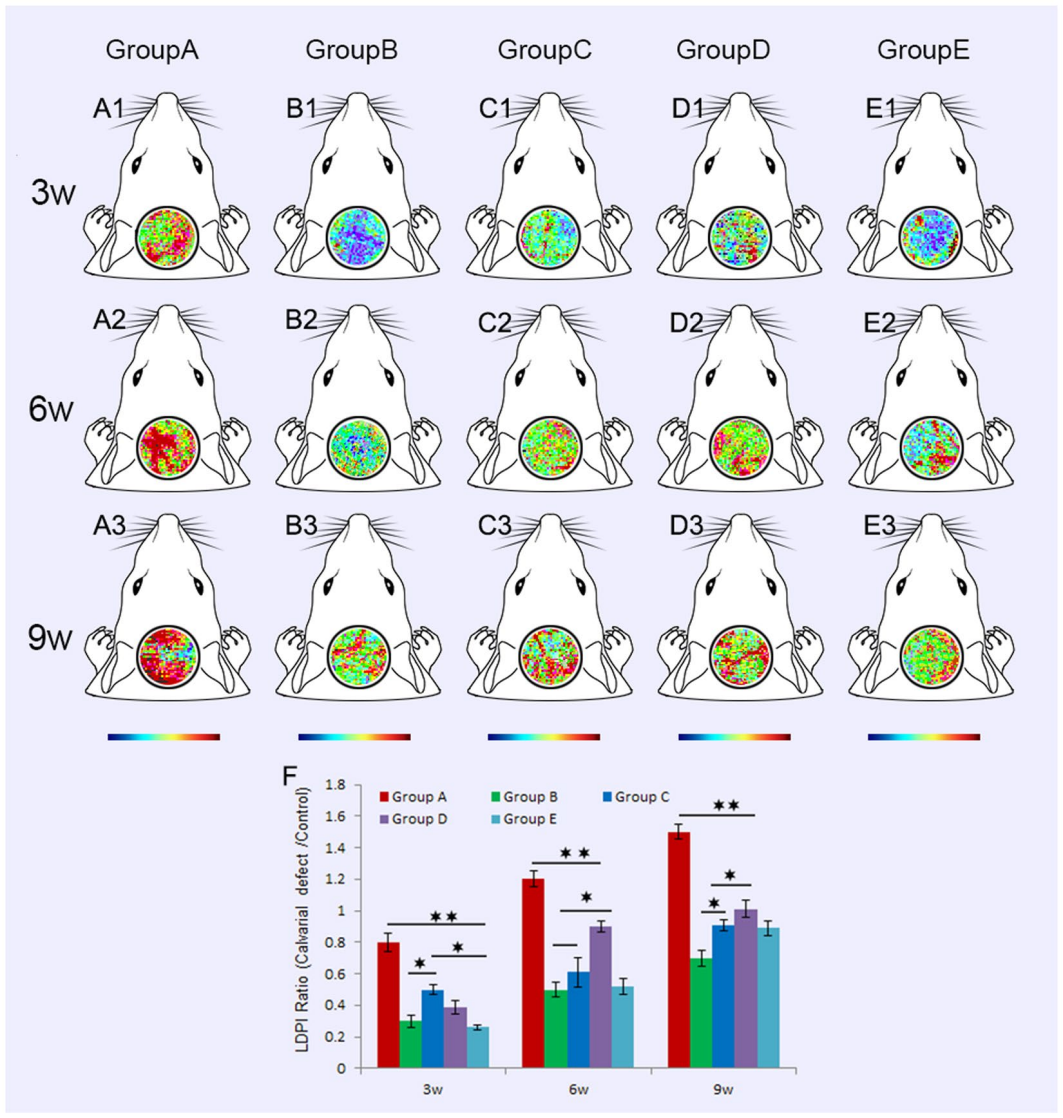
The time course of vascular perfusion was assessed using Laser Doppler imaging at 3, 6, and 9 weeks post operation. At 3 weeks, the degree of blood perfusion of Group A was higher than that in the other groups (p < 0.01). However, blood perfusion was significantly decreased as VEGF was inhibited. Lack of GDF15, microvascular blood flow was also decreased which was similar to the scaffold alone (**A1**–**E1**,**F**). At 6 and 9 weeks, blood perfusions of Group A was still higher than that of Group D (BMSCs/β-TCP), but both Group A and Group D were higher than the other three groups in reperfusion ratio at the two time points (**A2**–**E2**, **A3**–**E3**,**F**).

Laser Doppler imaging is a non-invasive method for monitoring of microvascular blood flow, a very important marker of regions of interest circulation. The time course of vascular perfusion was assessed at 3, 6, and 9 weeks post operation (Fig. 6). According to LDPI ratio analysis, at 3 weeks, the degree of blood perfusion of Group A was higher than that in the other groups (Fig. 6A1–E1). However, blood perfusion was significantly decreased as VEGF was inhibited (Fig. 6B1–B3). Lack of GDF15, microvascular blood flow was also decreased which was similar to the scaffold alone (Fig. 6C1–C3). At 6 and 9 weeks, blood perfusions of Group A was still higher than that of Group D (BMSCs/β-TCP), both Group A and Group D were higher than the other three groups in reperfusion ratio at the two time points (Fig. 6A2–E2;A3–E3). The results above demonstrated that GDF15 promoted neovascularization in the repair of rat critical size calvarial defect, which may be associated with VEGF.

### Analysis of neovascularization using micro-CT measurement

**Figure 7:**
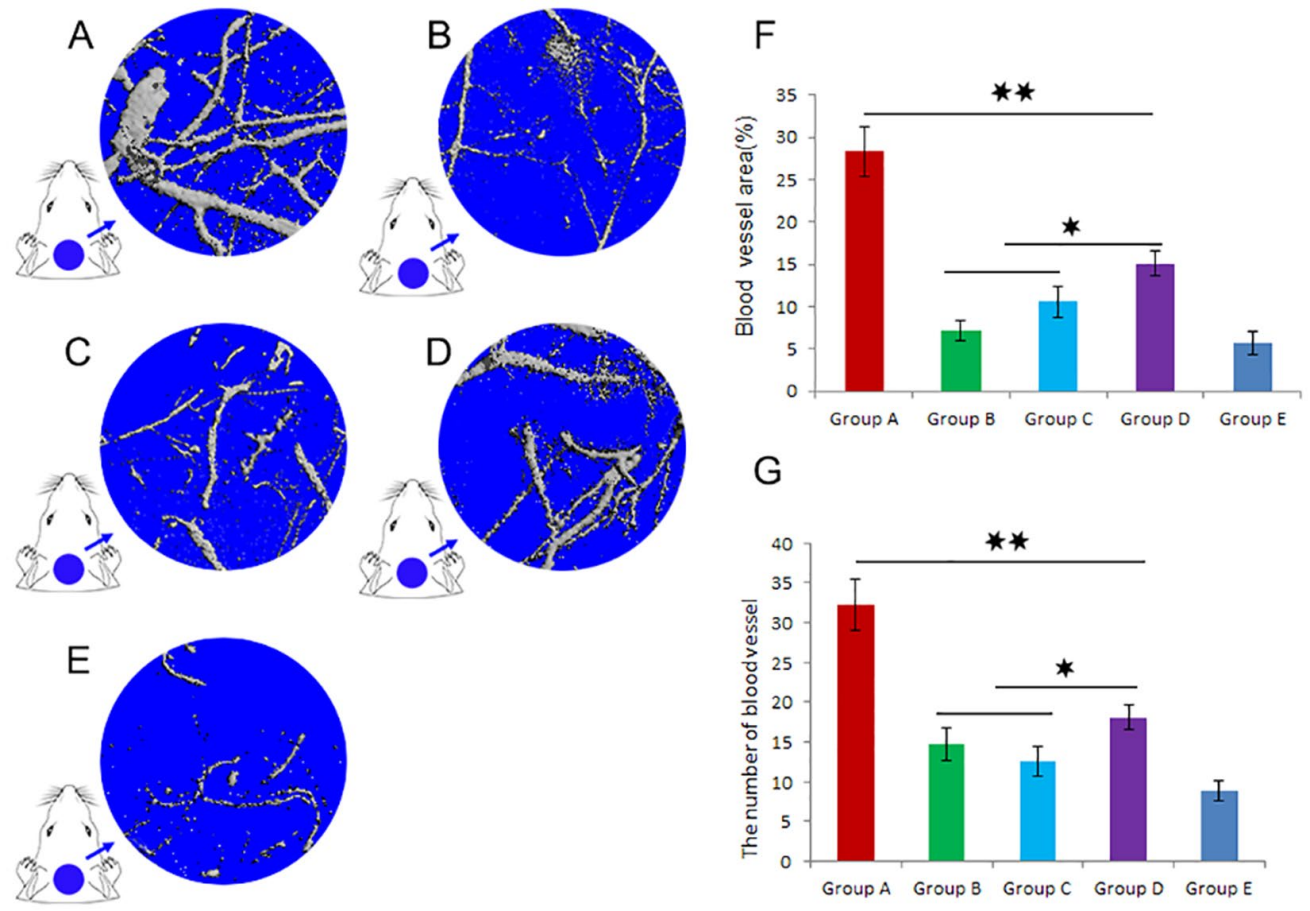
The effects of GDF15 promoting angiogenesis in critical sized calvarial defect were studied by microfil perfusing at 12 weeks post-surgery. The vascular network in the GDF15 group appeared more new blood vessels compared to the other five groups (**A**–**E**). Inhibiting VEGF could significantly decrease neovascularization in Group B. A significantly larger vascular network area was observed in the GDF15 group (**F**). The number of blood vessels in the GDF15 group was also higher than in the other groups (**G**) (P < 0.01).

The effects of GDF15 promoting angiogenesis on critical sized calvarial defect were examined by perfusing the vessels with microfil using micro-CT at 12 weeks post-surgery. The reconstructions of the three-dimensional micro-CT images directly revealed blood vessel formation in the bone defects (Fig. 7A–E). The vascular network in the GDF15-treated group showed more new blood vessels compared to the other five groups. Inhibiting VEGF could significantly decrease neovascularization in Group B. The detection of neovascularization in the defect sites was conducted using morphometrical quantification analysis. We found that there was a significantly larger vascular network area in the GDF15 group (Fig. F), and the number of blood vessels in the GDF15 group was also higher than in the other groups (Fig. 7G). The data revealed that GDF15 had a significant increase in the total vessel volume critical sized calvarial defect.

### Histological analysis

**Figure 8:**
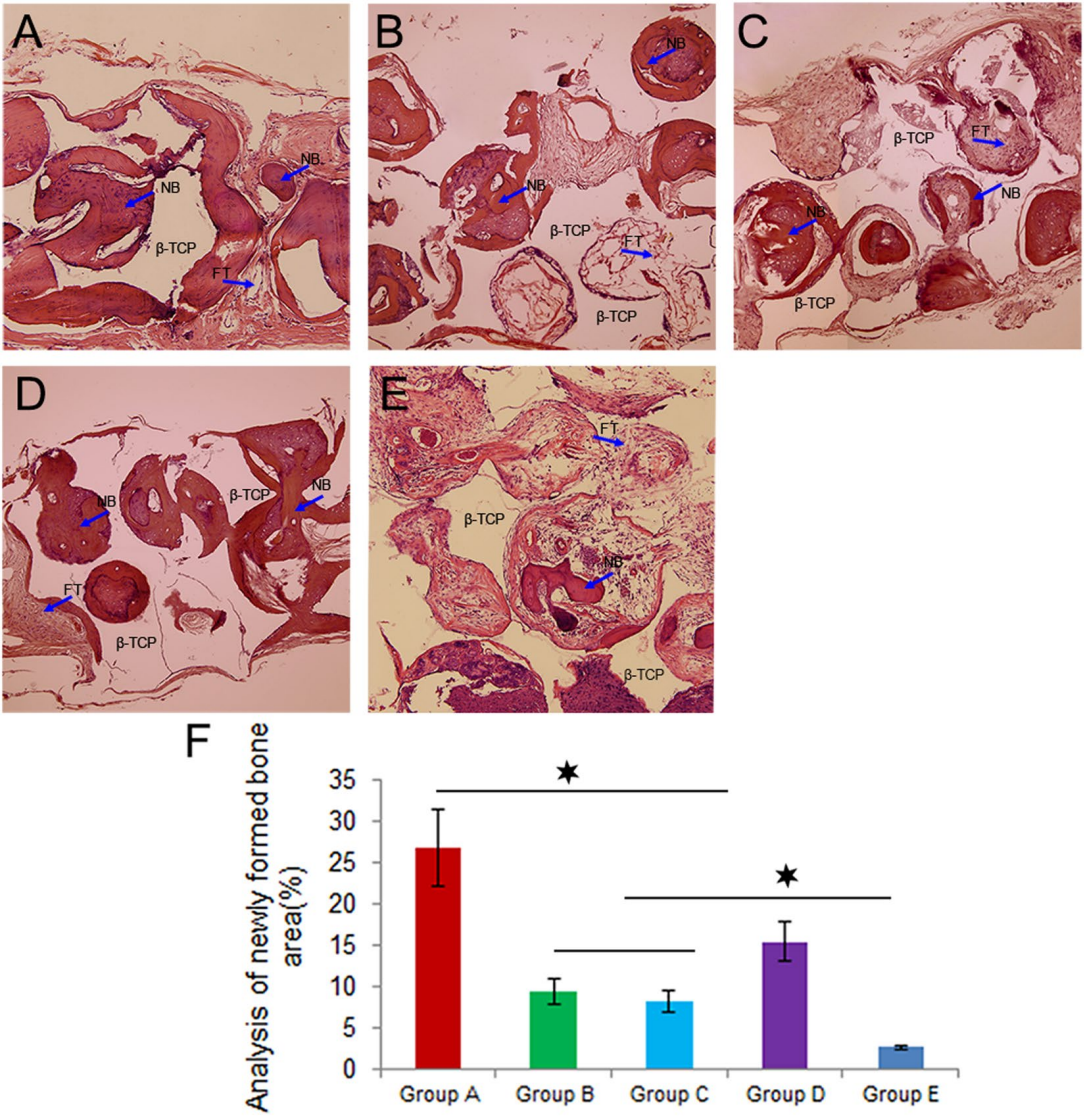
Under light microscopy, decalcified sections stained with HE showed that, in Group A, the area of newly-formed bone was the highest in five groups (**A**–**E**). Blue arrows show newly-formed bone (NB) or fibrous tissue(FT), and the empty space indicates β-TCP. Bone formation in Groups B and C was significantly lower than Group D (**F**).

Under light microscopy, decalcified sections stained with HE showed that Group A (GDF15/BMSCs/β-TCP) appeared to be the highest in the area of newly-formed bone in five groups (Fig. 8A–E). Though the area of bone formation in Group D(BMSCs/β-TCP) showed lower than that of Group A, it was still higher than that in Groups B(GDF15/Bevacizumab/BMSCs/β-TCP) and C (antiGDF15/BMSCs/β-TCP) (Fig. 8F). The newly formed bone in β-TCP alone was the lowest in the five groups.

## Discussion

New approaches to enhance vascularized tissue growth become one of the most active areas in regenerative medicine. Because of the close association between angiogenesis and osteogenesis, neovascularization is considered as an important element in the repair of bone defects. In this study, we demonstrated that GDF15 augmented the levels of cyclins D1 and E, increased protein kinase activity of CDKs and then enhanced the E2F-1 level. Additionally, we also observe the angiogenic capacity of GDF15 *in vitro* and vivo. These findings confirm that GDF15 promotes angiogenesis through stimulating endothelial cell proliferation, indicating that GDF15 may be a potential angiogenic cytokine in regenerative medicine.

GDF15, a divergent member of the TGF-β superfamily, has been implicated in many cellular processes, including angiogenesis^14, 15^, early bone formation^17^ and inflammation^9, 18^. A number of evidences show that GDF15 may be considered as a cardioprotective TGF-β superfamily protein, and serum GDF15 level become a biomarker for cardiac injury, which may provide an important prognostic information for heart disease^19–21^, Clinical study demonstrated that GDF15 was a biomarker of endothelial activation, however, the underlying mechanism remains unclear^22^. Reports have showed that VEGF could increase cell cycle progression, and then activate the proliferation of HUVECs^15^. Therefore, we infer that GDF15 promotes endothelial cell activation through the enhancement of cell cycle progression, and that it has a similar biological effects as VEGF in promoting angiogenesis^23, 24^. With respect to signaling pathways involved, Song H, *et al*. have shown that GDF-15 promoted angiogenesis possibly through inhibiting p53 signal, which subsequently enhanced and stabolized HIF-1alpha expression, and up-regulated the related downstream angiogenic signaling^25^. Nevertheless, the function of GDF15 needs to be further elucidated.

Rat critical size calvarial defect is an ideal animal model in regenerative medicine^16^. We previously applied the model in this study for evaluating the osteogenesis and angiogenesis^26^. β-TCP has good biocompatibility and osteoconductive capacity, which can be fabricated into high porosity scaffolds with good interconnectivity and ensure intercellular communication among osteogenic and angiogenic cells rested in lacunae. The macro-porosity of the material will facilitate cells adhesion and growth, and facilitate bone ingrowth and especially vascularization. They can be used for evaluating biological function of GDF15 in animal model.

In our study, we observed that GDF15 up-regulates expression of cyclins D1 and E in HUVECs. The increased expression levels of cyclins D1 and E resulted in enhanced protein kinase activity of CDK4-cyclin D1 and CDK2-cyclin E, respectively. Following Rb phosphorylation, E2F transcription factors were released from the E2F–Rb and induced the transcription of several genes involved in DNA replication. According to human phosphokinase array, we monitored that GDF15-treated HUVECs increased the phosphorylation levels of Akt, ERK1/2 and JNK kinases. These results indicate GDF15 may be involved in both pathways of Akt and MAP kinases including ERK and JNK to enhance cyclins D1 and E expression. As the proliferation of endothelial cell plays an important role in angiogenesis, GDF15 may be a potential angiogenic growth factor for tissue regeneration^23, 27^.

To further test the angiogenic effects of GDF15 on HUVECs, we next performed tube formation of endothelial cells in vitro Matrigel angiogenesis model, and found that GDF15 promoted branch points with the increasing concentration of GDF15. More importantly, *in vivo* the data showed that mice injected with Matrigel plugs supplemented with GDF15 had significantly more newly formed blood vessels in plugs compared to PBS groups. The ratio between Matrigel plug fluorescence and plasma fluorescence demonstrated that the highest ratio was 0.0024 ± 0.00018 in the 50 ng/ml group, as was comparable to VEGF group. We found no significant difference between 50 ng/ml and 100 ng/ml in the ratio, but both of them have more blood vessels than in 25 ng/ml group. The results from the Matrigel plug perfusion assay were consistent with Matrigel angiogenesis in vitro and Doppler and Microfil measurements in the healing of rat critical size calvarial defect. Based on Matrigel plug perfusion assay, we found that VEGF seemed to be more effective than GDF15 in angiogenesis. However, in this study, we didn’t find the decreased food intake and weight loss in all mice and rats^13^.

According to our observations in the repair rat criticize size calvarial defects, we found that a number of newly-formed blood vessels were induced by GDF15 in Group A, and blood vessels were significantly decreased when the administration of Bevacizumab or antibody to GDF15. The data indicated that elevated GDF15 levels at local region could stimulate neovascularization, which was reversed by administration of antibody to VEGF or GDF15. As for the mechanism of angiogenesis of GDF15 *in vivo*, we postulate that it may also be related with VEGF expression, because the administration of Bevacizumab can decrease blood vessels in Group B.

More contemporary studies have documented that osteogenesis was in close association with adjacent capillary ingrowth, and impairment of angiogenesis decreased bone formation^3^. Based on decalcified sections, we found that the area of newly-formed bone in calvarial defects was the highest in Group A (GDF15/BMSCs/β-TCP) than that of the other four groups. Bone formation in Group B and Group C, with the consecutive administration of antibody to VEGF or GDF15, was significantly lower than Group A. The reason may be that newly formed blood vessels provided the implanted cells with oxygen, nutrients, and soluble factors, as a result, promoted new bone formation in the region of bone defects. By comparsion, lack of sufficient blood vessels formation, the newly formed bone in Group E was the lowest in the five groups.

In conclusions, the present study demonstrates that GDF15 stimulates endothelial cell proliferation through the increase of cyclins D1 and E, CDKs and E2F-1 expression, and promotes angiogenesis. The signaling pathway involved may include Akt, ERK1/2, and JNK. The angiogenic activity of GDF15 was confirmed by Matrigel plugs array and animal model of rat critical-sized calvarial defect. Our results indicate that GDF15 may be a potential angiogenic cytokine and it can be applied for promoting vascularization in regenerative medicine.

## Materials and Methods

### Cell culture

HUVECs were purchased from Chinese Academy of Sciences and cultured in M200 medium supplemented with 20% fetal bovine serum (FBS), 100 U/ml penicillin, 100 μg/ml streptomycin, 5 U/ml heparin in humidified 5% CO_2_ at 37 °C. Rat BMSCs were isolated and cultured according to our previous protocol^28^. Cells at passage 2–3 were used for the following experiments. All procedures concerning animal use were approved by the Animal Research Committee of the Ninth People’s Hospital which is affiliated with Shanghai JiaoTong University Medical School (Shanghai, China).

### Cell proliferation assay

HUVECs proliferation was determined by MTT^29^. Briefly, HUVECs at a density of 6.0 × 10^3^ cells/well were cultured in a 96-well plate for 6 h with M200 medium containing 1% FBS and treated with GDF15 or VEGF for 24 h. MTT (Sigma, St Louis, MO, USA) solution was added into each well, and cells were incubated for 3 h to form formazan. Formazan crystals were dissolved in dimethyl sulfoxide (DMSO) and measured at 490 nm using ELX Ultra Microplate Reader (Bio-Tek, Winooski, VT, USA). All experiments were done in six replicates (n = 6).

### Cell cycle analysis

HUVECs grown to 80% confluency were serum-starved in 1% FBS-containing M200 medium for 12 h, and treated with or without GDF15 for another 12 h. Following treatment, cells were released to enter the cell cycle with the addition of M200 complete medium containing 20% FBS for 12 h. The cell growth cycle was examined using propidium iodide (PI). 1.0 × 10^6^ cells were harvested by centrifugation, then fixed in ethanol and stained with PI/RNase (Sigma, USA). The samples were analyzed using FASC Calibur flow cytometer (Beckman Coulter Company, USA). Percentages of cells existing within the various phases of the cell cycle were calculated using Cell Quest by gating on G0/G1, S, and G2/M cell populations.

### Real-time PCR and Western blotting analysis

Total cellular RNA was purified from cultured cells using TRIzol reagent (Invitrogen) according to the manufacturer’s protocol. Reverse transcription was carried out using 1 μg of total RNA in a final volume of 20 μL using a PrimeScript RT reagent kit (Takara Bio, Shiga, Japan). The realtime PCR was performed and the relative expression of each target mRNA was calculated. Gene-specific primers were synthesized commercially (Shengong Co. Ltd. Shanghai, China)**:** 5′-CTGTCCTA CTACCGCCTCAC-3′ (forward) and 5′-ACCTCCTCCTCCTCCTCTTC-3′ (reverse) for Cyclin D1; 5′-CTGGATGTTGACTGCCTTGAAT-3′ (forward) and 5′-TTCTCTATGTCGCACCACTGA-3′ (reverse) for Cyclin E; 5′-CGTGCGTG ACATTAAGGAGAA-3′ (forward) and 5′-GGAAGGAAGGCTGGAAGAGT-3′ (reverse) for β-actin. All mRNA values were normalized against β-actin expression.

For western blotting, proteins were isolated and quantified using the BCA method(Pierce, Rockford, IL). For each sample, 30 μg protein was loaded onto a 10% SDS–polyacrylamide gel. After electrophoresis, protein was transferred onto a PVDF membrane (Millipore, MA, USA) by electro-elution. The membrane was incubated initially with a specific primary antibody in Tris-buffered saline (TBS) containing 0.05% Tween 20 (TSB-T) and 5% nonfat dry milk at 4 °C overnight, then with HRP-conjugated goat anti-Rabbit IgG (Cell signaling) for 2 h at room temperature. After washing, the membrane was processed using Immobilon™ Western chemiluminescent HRP substrate (Millipore). β-Actin (Cell signaling) was served as internal control.

### Co-immunoprecipitation

Cell pellets were suspended in Tris-buffer containing 1% (vol/vol) Triton X-100 and 0.5% (wt/vol) sodium dodecyl sulfate (SDS) at 4 °C. After centrifugation at 10,000 g for 20 min, the cell extracts (250 μg protein) in a final volume of 500 μl were precleared for 30 min with 1.0 μg of non-immune IgG. Twenty microliters of a 50% slurry of Protein A/G sepharose beads (Invitrogen) were added, and after 30 min of incubation, the solution was centrifuged as above. Supernatants were incubated for 2 h with 20 μl of antibody against human CDK2 (Abcam), or CDK4 (Abcam) or Rb (Abcam) at 4 °C, after which 50 μl of a 50% slurry of A/G beads were added, and the solution was incubated for an additional hour. Then the tube was placed on the magnet and transfered the supernatant to a clean tube. After washed the Dynabeads-Ab-Ag complex 3 times, we resuspended the complex in 100 μL Washing Buffer and transfered the bead suspension to a clean tube. Then proceeded to elute target antigen, and immunoblots were performed using antibodies diluted in 1% BSA TBST. Anti-Histone H1 (1:1,000 dilution), anti-E2F (1:1000 dilution) were used. Secondary antibodies (Cell signaling) were used to visualize immunoblots. Images were captured and the density of each band was analyzed with GelDoc software (Bio-Rad, Munich, Germany).

### Human phosphorkinase array

HUVECs were treated with 25,50, and 100 ng/mL rhGDF15 for 1 h, and total protein was purified. Non-treated- HUVECs were used as control. Cell lysates (250 mg total protein per array) were applied and incubated with Human phospho-kinase array kit (R&D Systems, MN, USA) according to the manufacturer’s instructions. Gray values were quantified using NIH image software analysis^30^.

### *In Vitro* HUVEC Tube Formation Assay

Matrigel was distributed in a 96-well plate (60 μl/well) and allowed to solidify at 37 °C.HUVECs were serum-starved in M200 medium for 6 h and seeded at 1.0 × 10^4^ HUVECs/well. The cells were cultured in M200 medium, with 25 ng/ml, 50 ng/ml, and 100 ng/mL at 37 °C for 12 h, 24 h, and 48 h. Then we carefully removed medium and added 2 µg/mL cell‑permeable dye solution (Calcein AM, Sigma, USA) per well to make the network more visible. Tube formation was observed using a Confocal Laser Scanning Microscope (CLSM, Leica TCS Sp5 Germany, Excitation/Emission wavelength was 488/517 nm)^31^. Finally, the fluorescence images were analyzed using the Image pro Plus 6 software to quantitate the extent of tube formation by counting the capillary tube branch points formed in each replicate well.

### *In vivo* angiogenesis assessment by Matrigel plug perfusion assay

Our next objective was to determine if these *in vitro* observations were accordance with the effects *in vivo*. Then Matrigel plug assay was performed in Balb/c mice^32^. Briefly, 0.5 ml Matrigel (Becton, Dickinson and Company, USA, 354252) was injected subcutaneously with 4-week-old male mice in the ventral groin area, which was supplemented with 25 ng/ml, 50 ng/ml, 100 ng/ml GDF15, and PBS alone and VEGF were used as control. On day 14, 0.2 mL of 25 mg/mL FITC-dextran in PBS was injected into tail vein 30 min before being sacrificed. Blood samples were collected, and plasma was separated and protected from light. Matrigel plugs were harvested and placed into tubes containing 1 mL of 1:10 dispase, and incubated in the dark in a 37 °C shaker overnight. Then the matrigel plugs were homogenized and centrifuged at 16000 rpm for 15 min. Supernatants were collected and stored in the dark at 4 °C. Supernatant fluorescence was measured in a fluorometer (Synergy™ HT, Multi-Detection Microplate Reader, BIO-TEK Instruments, Inc. Winooski, VT, USA), and angiogenic response was expressed as a ratio of Matrigel plug fluorescence/plasma fluorescence^33^.

### Animal model for angiogenesis of cranial defect in rat

Surgical procedures were performed on six-week-old male Fisher 344 with a weight of 220 g ± 15 g, as previously described^26^. Briefly, the animals were anesthetized by intraperitoneal injection of pentobarbital (Nembutal 3.5 mg/100 g). A 1.5 cm sagittal incision was made on the scalp, and the calvarium was exposed by blunt dissection. One critical-sized bone defects were created by a 8-mm diameter trephine burr (Kavo, K5plus, Germany). BMSCs/β-TCP constructs or β-TCP were placed in the defects, and the incision was closed (Figures S4 A and B).

Preparation of BMSCs/β-TCP constructs: β-TCP scaffolds (diameter: 8mm, thickness: 1.5 mm, Shanghai Bio-Lu Biomaterials Co. Ltd., Shanghai, China) had volume porosity of 70% with average pore diameter of 450 ± 50μm. For cell seeding, BMSCs were detached from culture dishes, centrifuged to remove supernatant, and then resuspended in the culture media without FBS at a density of (20 × 10^6^cells/ml). Cells in suspension were slowly added to β-TCP till a final saturation. After incubation for an additional 4 h to allow cell attachment, the scaffolds were implanted in the defects for six rats per group. In a parallel experiment, scaffolds were prepared and seeded with BMSCs at an identical cell density. Four and 24 h later, the constructs were fixed in 2% Glutaric dialdehyde for 2 h, and then subjected for SEM examination (Philips SEM XL-30, Amsterdam, Netherlands) (Figures S4 C and D).

Animals received local administration of Vehicle (sterile saline), or GDF15 (2 μg, R&D, Minnesota, USA), or antibody to GDF15 (2 μg, Abcam, Cambridge, UK), or Bevacizumab (2 μg, Genentech/Roche, San Francisco, CA, USA) thrice a week for three months^34–36^. Samples were harvested 12 weeks after implantation. After the animals were sacrificed, cranial samples were perfused with Microfil (Flowtech, Carver, MA) after euthanasia to evaluate blood vessel as previously described^37^. Five groups include: Group A: GDF15/BMSCs/β-TCP (n = 6); Group B: GDF15/Bevacizumab/BMSCs/β-TCP (n = 6); Group C: antiGDF15/BMSCs/β-TCP (n = 6); Group D: BMSCs/β-TCP (n = 6); Group E: β-TCP alone (n = 6).

### Laser Doppler imaging to assess blood perfusion at different times

Laser Doppler imaging observation was performed as our previous report^38^. At 3, 6, and 9 weeks post operation, animals were anesthetized for physiological evaluation of microcirculation using a laser Doppler perfusion imager (LDI, Moor Instruments, Devon, UK). Digital color-coded images were analyzed using the LDPI image analysis software to quantify the blood flow in the implantation site.

### Microfil perfusion and micro-CT measurement

To evaluate blood vessel formation, 12-week rats were perfused with Microfil (Flowtech, Carver, MA, USA) as previously described^26^. Briefly, animals were anesthetized and perfused with 20 ml of 37 °C PBS plus 10 units/ml heparin at a flow rate of 10 ml/min through the left ventricle. After PBS, rats received 20 ml of 4% paraformaldehyde, and 20 ml of Microfil. Finally, the samples were set overnight at 4 °C and demineralized using a 10% formic acid solution prior to scanning. The blood vessel area and the vessel number were analyzed by micro-CT and Image Pro 5.0 (Media Cybernetics, USA).

### Histological observation

The samples were then fixed in 10% buffered formalin, then decalcified and embedded in paraffin, sectioned into 4 μm thick sections, and stained with hematoxylin–eosin. The measurements were performed using a personal computer- based image analysis system (image-Pro Plus, Media Cybernetic, Silver Springs, MD, USA). Three randomly selected sections from the serial sections collected from each sample were analyzed.

### Statistical Analysis

Results are presented as mean ± SD. Statistical significance was assessed by ANOVA with a SNK post hoc. *P* < 0.05 was considered statistically significant. (*P < 0.05 and **P < 0.01, the experimental groups comparing with the control group). Statistical analysis was performed using a SAS 6.12 statistical software package (Cary, NC, USA).

## Electronic supplementary material

supplementary data
