# Supplementary material for: Growth differentiation factor 15 promotes blood vessel growth by stimulating cell cycle progression in repair of critical-sized calvarial defect
Source: Sci Rep. 2017 Aug 22;7:9027. doi: 10.1038/s41598-017-09210-4 (PMC5567281; doi:10.1038/s41598-017-09210-4)

Supplementary information

Growth differentiation factor 15 promotes blood vessel growth by stimulating cell cycle progression in repair of critical-sized calvarial defect

Shaoyi Wang[1], Mengyu Li[1], Wenjie Zhang[2,4], Hongfei Hua[1], Ningtao Wang[1], Jun Zhao[3], Jing Ge[1], Xinquan Jiang[2,4], Zhiyuan Zhang [1], Dongxia Ye[5] & Chi Yang [1]

[1]Department of Oral Surgery, Ninth People's Hospital, Shanghai Jiao Tong University School of Medicine, Shanghai Key Laboratory of Stomatology, National Clinical Research Center of Stomatology

[2]Department of Prosthodontics, Ninth People's Hospital, Shanghai Jiao Tong University School of Medicine, Shanghai Key Laboratory of Stomatology, National Clinical Research Center of Stomatology

[3]Department of Orthodontics, Ninth People’s Hospital, Shanghai Jiao Tong University School of Medicine, Shanghai Key Laboratory of Stomatology, National Clinical Research Center of Stomatology

[4]Oral Bioengineering Lab/Regenerative Medicine Lab, Shanghai Research Institute of Stomatology, Ninth People's Hospital, Shanghai Jiao Tong University School of Medicine, Shanghai Key Laboratory of Stomatology, National Clinical Research Center of Stomatology

[5]Shanghai Research Institute of Stomatology, Ninth People's Hospital, Shanghai Jiao Tong University School of Medicine, Shanghai Key Laboratory of Stomatology, National Clinical Research Center of Stomatology

Correspondence and requests for materials should be addressed to Z.Z.Y (email: zhzhy@omschina.org.cn), Y.D.X (email:yedongxia122@hotmail.com), or Y.C (email: yangchi63@hotmail.com)

Figure legends:

Figure S1. Schematic diagram of GDF15 promoting angiogenesis in vitro and in vivo.

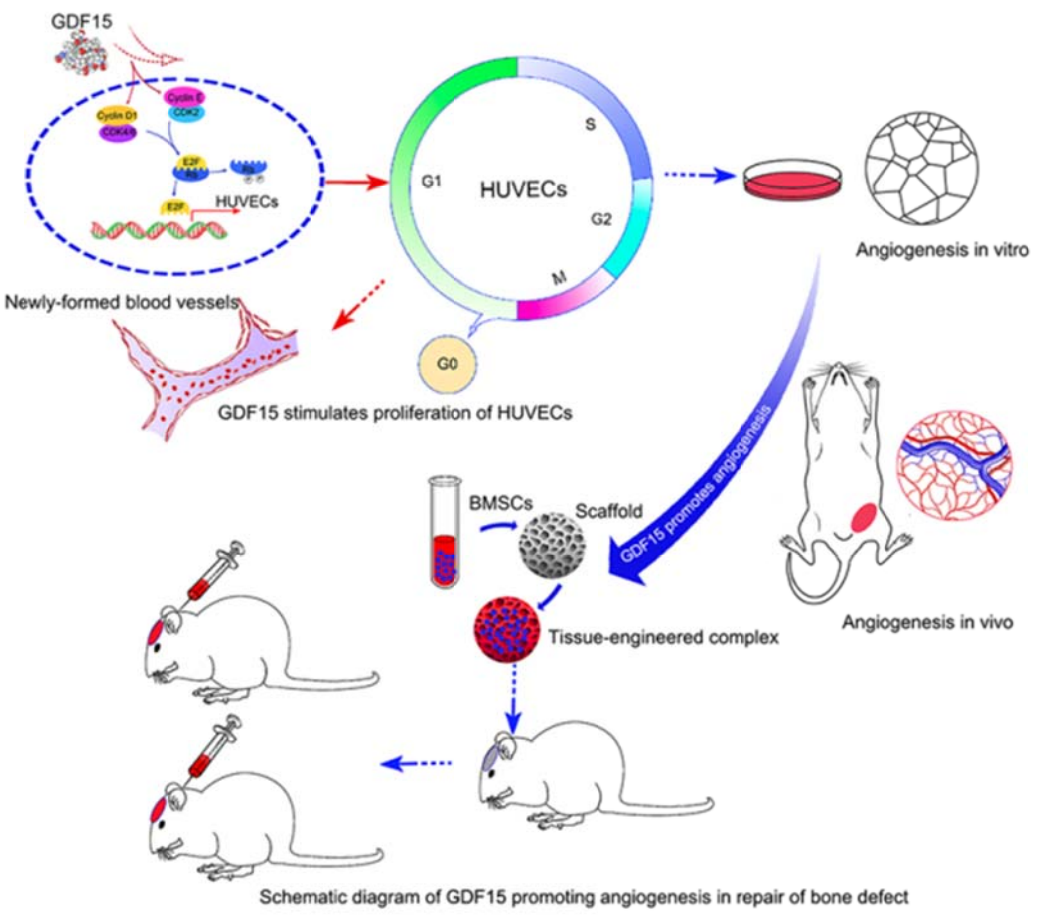

Figure S2. The effect of GDF15 on HUVECs proliferation was determined by MTT. HUVECs were treated with rhGDF15 for 24h, and cell proliferation was increased in a dose dependent manner.

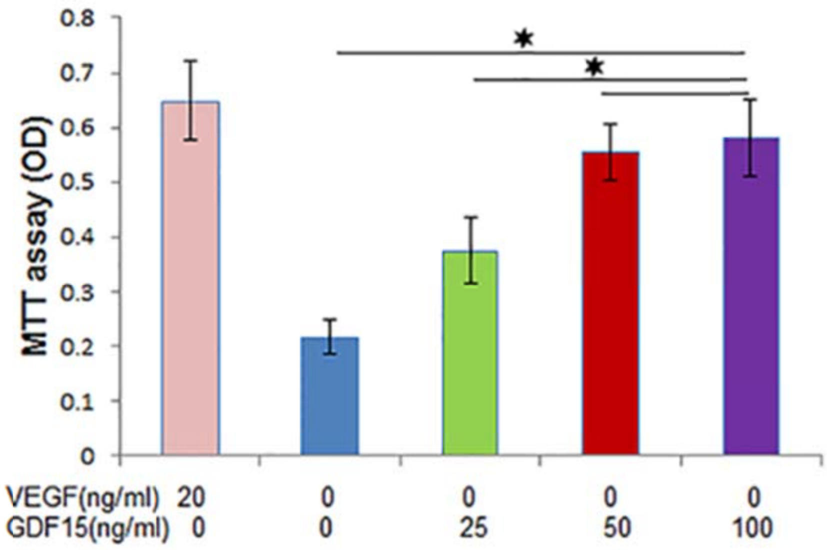

Figure S3. Signaling pathways regulated by GDF15. Phosphokinase array for GDF15-stimulated HUVECs with the concentration of 0, 25, 50, or 100ng/ml. Plates A and B were incubated with 300 μg of cell lysate. Protein profiling in the array was presented as pixel density(A). Cell lysates were probed for phosphorylated AKT, ERK, and JNK, and the results were expressed in relative units (phosphorylated protein/total protein) (B).

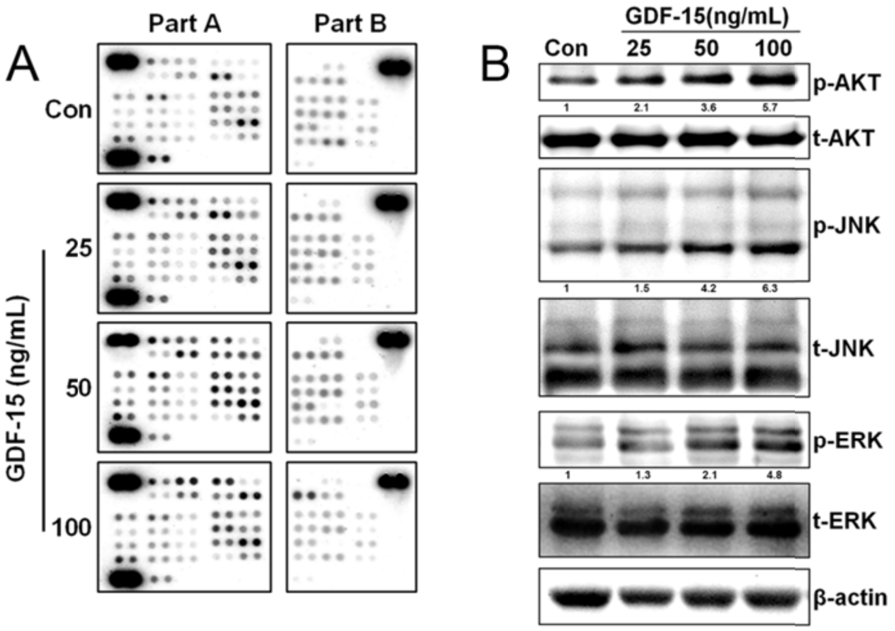

Figure S4. Animal model of rat critical size calvarial defect (A). The complex of β-TCP/rBMSCs was implanted into calvarial defect (B). Scanning electron microscopic evaluation for three-dimensional porous structures of β-TCP (C), rBMSCs spread on the surface of the scaffold at 24 h after combining with β-TCP (D).

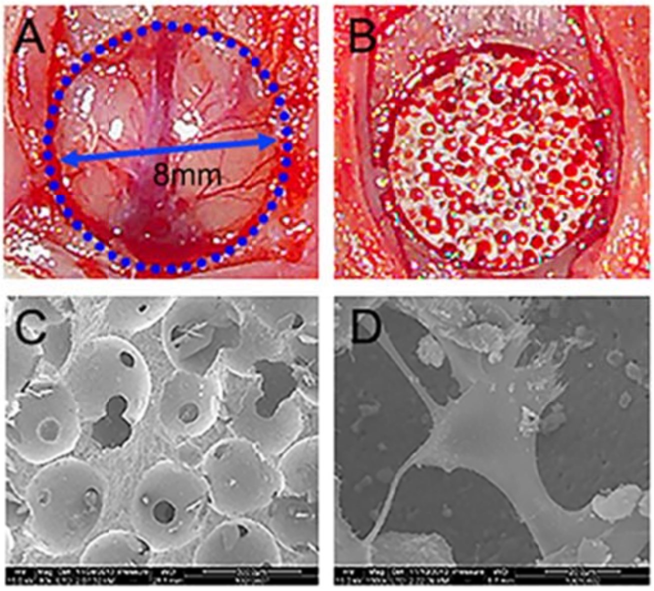

Supplement: Supplementary file 1 — supplementary data [file 41598_2017_9210_MOESM1_ESM.pdf]
